# Surviving without BRCA2: MLH1 gets R-looped in to curtail genomic instability

**DOI:** 10.1172/JCI179325

**Published:** 2024-04-01

**Authors:** Neil Johnson

**Affiliations:** Fox Chase Cancer Center, Nuclear Dynamics Program, Philadelphia, Pennsylvania, USA.

## Abstract

While breast cancer 2 (*BRCA2*) loss of heterozygosity (LOH) promotes cancer initiation, it can also induce death in nontransformed cells. In contrast, mismatch repair gene mutL homolog 1 (*MLH1*) is a tumor-suppressor gene that protects cells from cancer development through repairing mismatched base pairs during DNA mismatch repair (MMR). Sengodan et al., in this issue of the *JCI*, reveal an interplay between the 2 genes: MLH1 promoted the survival of BRCA2-deficient cells independently of its MMR function. MLH1 protected replication forks from degradation, while also resolving R-loops, thereby reducing genomic instability. Moreover, MLH1 expression was regulated directly by estrogen, shedding light into the hormone-responsive nature of many *BRCA2* mutant breast cancers. These results provide important insight into the genetics that drive the initiation of *BRCA2*-mutated breast cancers.

## MLH1 to the rescue

Breast cancer 1 (*BRCA1*) and *BRCA2* loss of heterozygosity (LOH) is a paradigmatic event in cancer initiation. However, the relationship between *BRCA1/2* genetics and cellular fitness represents an unresolved paradox, with *BRCA1/2* loss inhibiting the viability of normal cells yet promoting the development of cancer (1, 2). This quandary leads one to formulate the logical hypothesis that cancer cells must first, or at least simultaneously, acquire accompanying epigenetic and/or genetic modifications that enable normal cells to survive without BRCA2 and eventually transition to malignancy. Currently, the identity, timing, and crosstalk between potential genetic modifications leading to *BRCA1/2* mutation–associated cancer initiation have remained elusive. In large part, difficulty modeling the natural progression of disease stems from the fact that mice heterozygous for *Brca1/2* mutations, unlike humans, are not cancer prone (3).

In a study published in this issue of the *JCI* (4), the Sharan laboratory leveraged an elegant genetic system to provide mechanistic insights into the above questions. In previous work, this group established a PL2F7 mouse embryonic stem cell (mESC) line that contains a *Brca2* conditional knockout allele, termed *Brca2^cKO^* (1). In this case, Cre-induced recombination between *loxp* sites produced a null allele, which was unable to produce any BRCA2 protein product. The advantageous feature of this approach is the production of an intact *HPRT* minigene that allows for the selection of BRCA2-null cells when grown in hypoxanthine-aminopterin-thymidine (HAT) media. While *Brca2^KO/KO^* is lethal, the group previously discovered that pretreatment with the MRE11 inhibitor mirin can rescue the viability of a subpopulation of *Brca2^KO/KO^* cells (1). However, the mechanism was poorly defined. In Sengodan et al., the authors characterized mirin-rescued cells, referred to as *Brca2^KO/KO-r^* mESCs (4). Assessment of gene expression profiles revealed that several mismatch repair (MMR) complex proteins were overexpressed in independent *Brca2^KO/KO-r^* clones. However, only mismatch repair gene mutL homolog 1 (*Mlh1*) silencing blocked the capacity of mirin to rescue viability (4). Because silencing of other MMR proteins did not affect viability, MLH1 likely functions via a mechanism unrelated to the repair of mismatched base pairs (5).

## MMR-independent MLH1 activity

BRCA2’s ability to support homologous recombination (HR) was recently established as essential for genome stability and cell and organismal viability (6). In contrast, a separation-of-function mutation, which specifically disrupted DNA replication fork (RF) protection and gap suppression, had limited impact on genome stability. This finding led Sengodan and colleagues to initially ask whether MLH1 restored HR in *Brca2^KO/KO-r^* cells. However, cells lacked RAD51 foci and were highly sensitive to PARPi. The authors concluded that *Brca2^KO/KO-r^* cells were HR defective and that MLH1 supported viability via alternative mechanisms (4).

BRCA2-deficient cells are frequently defective in their ability to protect stalled DNA RFs from nuclease-mediated degradation (7). Surprisingly, *Brca2^KO/KO-r^* cells did not show RF degradation following hydroxyurea treatment. However, when MLH1 was silenced, DNA2-dependent fork degradation occurred, indicating that MLH1 was required to protect stalled RFs from DNA2-mediated degradation. In the setting of HR proficiency, the fork protection (FP) function of BRCA2 has limited impact on genome stability and cell viability (6). However, it is unclear whether RF protection effects cellular fitness when HR is defective. Whether the RF protection function of MLH1 affected the viability of BRCA2-null cells was not dissected and would be important to address in future work.

**Figure 1:**
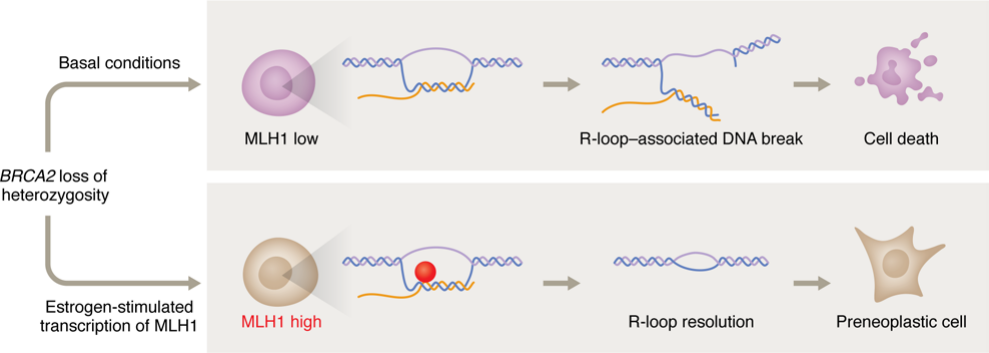
MLH1 limits genomic instability in breast epithelial cells with *BRCA2* LOH. *BRCA2* LOH results in the accumulation of unresolved R-loops. Under basal conditions, R-loops devolve into DNA breaks that induce cell death. In contrast, estrogen stimulated the transcription of MLH1, which directly resolves R-loops by processing the RNA strand. The resolution of R-loops prevents replication-transcription collisions and reduces overall levels of genomic instability, allowing preneoplastic cells to survive and continue through the stages of malignancy.

BRCA2 deficiency is known to result in an accumulation of R-loops, which in turn is a source of genomic instability (8). Using PCNA-S9.6 proximity ligation assay (PLA) foci to measure replication-associated R-loops, MLH1 silencing was found to increase foci in *Brca2^KO/KO-r^* cells. Interestingly, MLH1 degraded the RNA strand of the R-loop structure in in vitro nuclease assays, suggesting that it acts directly upon and resolves R-loops. Moreover, H2AX-S9.6 PLA foci increased when MLH1 was silenced. These data indicate that MLH1 likely resolved R-loops at RFs, and in the absence of MLH1, replication-transcription collisions devolved into DNA breaks. Importantly, the role of MLH1 in resolving R-loops was linked directly to its role in supporting the viability of BRCA2-null cells. Here, overexpression of *Rnaseh1* reduced R-loops and rescued the reduction in cell viability in MLH1-silenced BRCA2-null cells. These results suggest that R-loop resolution is essential for the viability of BRCA2-null cells (Figure 1).

## Importance to cancer

The identification and validation of synthetic lethal relationships offer opportunities for the development of anticancer therapeutic strategies. To cement the BRCA2/MLH1 synthetic lethal relationship, mouse genetic experiments were performed. *Brca2^L2431P/L2431P^* mice are born at reduced Mendelian frequencies, owing to the hypomorphic BRCA2 L2431P protein, while *Mlh1^KO/KO^* are also viable but infertile. Intercrossing double-heterozygous mice revealed the *Brca2^L2431P/^ ^L2431P^*, *Mlh1^KO/KO^* genotype was embryonic lethal. Moreover, mouse embryonic fibroblasts (MEFs) derived from *Mlh1^KO/KO^* mice exhibited a dramatic increase in R-loops, further supporting the role of MLH1 in suppressing R-loops as a source of genomic instability and the driver of lethality. These results underscore that MLH1 expression is not only imperative for BRCA2-null cells, but also essential to cells that express BRCA2 hypomorphic proteins and retain residual HR. Of note, the *Brca2^L2431P^* mutation disrupts the BRCA2-DSS1 interaction. Thus, it will be of interest to determine whether there are *BRCA2* mutations and functions that show hyper or reduced MLH1 dependencies.

Additional experiments in Sengodan et al. (4) focused on targeting MLH1 in cancer cells. Specifically, MLH1 shRNA delayed xenograft formation and growth from KBP1.21 BRCA2-null cancer cells relative to wild-type controls, indicating that MLH1 may also be a useful target in established cancers. Intriguingly, data from The Cancer Genome Atlas (TCGA) demonstrated that the fraction of the genome with alterations was higher when expression of both *BRCA2* and *MLH1* was low. This relationship was not observed with *BRCA2* and *MLH3*. Moreover, in cancer samples with high microsatellite instability, only 12% of samples with *MLH1* mutation showed *BRCA2* mutations, while 26% of samples with *PMS2/MSH6/MSH2* mutations had *BRCA2* mutations. These analyses, when combined with genetic experiments, firmly establish the MMR-independent nature of the BRCA2-MLH1 synthetic lethal relationship.

Based on positive immunohistochemical staining for the estrogen receptor α (ERα) in many *BRCA2* mutant breast cancers (9), the authors investigated the potential interplay between ERα signaling and MLH1 (4). MLH1 expression was higher in luminal compared with basal subtypes of breast cancer at the RNA and protein levels and directly correlated with ERα expression. Moreover, an estrogen-responsive element (ERE) was present in the promoter of MLH1 that showed the expected outcomes in response to tamoxifen versus estrogen. These experiments give credence to the hypothesis that an ERα/MLH1 axis aids BRCA2-deficient breast epithelial cells in their traversal to malignancy.

## Conclusions and future directions

The immediate genomic instability ensuing from LOH of *BRCA1/2* restrains carcinogenesis due to its deleterious impact on cell viability. However, cooperating events that facilitate the transition of *BRCA2* mutation–containing cells to cancers typically prevail; their identity and mechanisms of action are slowly emerging. TP53, TET2, PARP1, MRE11, and now MLH1 have been shown to contribute to cell survival in the absence of BRCA2 (1, 10). Whether there is functional interplay between these proteins or overlapping activities, perhaps central to preserving RF fidelity, remains to be determined. In response to mismatched base pairs, MLH1 heterodimerizes with PMS2, and endonuclease activity generates single-stranded DNA breaks. MLH1 is now shown to protect the RF from degradation as well as resolve R-loops that accumulate when BRCA2 is absent. The MLH1 protein domains, regions, and specific functions required for each of these activities have not been identified and are of interest for investigation in future studies.

MLH1 directly degraded the RNA strand of R-loops in vitro, but how MLH1 operates in cells in conjunction with other R-loop–processing factors, especially given no other MMR factors affected BRCA2-deficient cell viability, is of interest and yet to be determined. Presumably, canonical R-loop–processing proteins will be more active in BRCA2-null cells, either at the transcriptional or posttranslational level. Given R-loop processing is key for survival, it is unclear why more regulators of R-loops are not synthetic lethal with BRCA2. Perhaps the dual role of MLH1 in preserving RFs that stall as they encounter R-loops provides them with the specific capability to deal with transcription-replication collisions. Of note, BRCA1-deficient cells also accumulate R-loops (11), and it will be of interest to examine the role of MLH1 in *BRCA1* mutant cancers.

Finally, Sengodan, et al. (4) highlight the genetic complexities that orchestrate the transition from normal tissue to disease states as well as the pleiotropic nature of DNA-repair proteins. While HR-proficient cells rely on MLH1 to repair mismatched bases, HR deficiency induces a requirement for MLH1 in resolving R-loops. Of note, *BRCA2* heterozygous cells also have RF defects (6). Determining whether defective RFs create a selection pressure for *MLH1* overexpression prior to *BRCA2* LOH would be of importance. Drug-development approaches targeting proteins that are crucial for survival as normal cells undergo *BRCA1/2* LOH could provide promise, particularly for early cancer interception among high-risk mutation carriers, and warrant future exploration.

## Version 1 04/01/2024

Electronic publication
